# Environmental factors involved in SARS-CoV-2 transmission: effect and role of indoor environmental quality in the strategy for COVID-19 infection control

**DOI:** 10.1186/s12199-020-00904-2

**Published:** 2020-11-03

**Authors:** Kenichi Azuma, U Yanagi, Naoki Kagi, Hoon Kim, Masayuki Ogata, Motoya Hayashi

**Affiliations:** 1 Department of Environmental Medicine and Behavioral Science, Faculty of Medicine, Kindai University, 377-2 Ohnohigashi, Osakasayama, 589-8511 Japan; 2 Department of Architecture, School of Architecture, Kogakuin University, Tokyo, 163-8677 Japan; 3 Department of Architecture and Building Engineering, School of Environment and Society, Tokyo Institute of Technology, Tokyo, 152-8550 Japan; 4 Department of Environmental Health, National Institute of Public Health, Wako, 351-0197 Japan; 5 Department of Architecture and Building Engineering, Tokyo Metropolitan University, Tokyo, 192-0397 Japan; 6 Laboratory of Environmental Space Design, Division of Architecture, Faculty of Engineering, Hokkaido University, Sapporo, 060-6826 Japan

**Keywords:** Air quality control, COVID-19, Environmental factor, Indoor environment, Precautionary approach, SARS-CoV-2, Pathway, Ventilation

## Abstract

The severe acute respiratory syndrome coronavirus 2 (SARS-CoV-2), a new zoonotic agent that emerged in December 2019, causes coronavirus disease 2019 (COVID-19). This infection can be spread by asymptomatic, presymptomatic, and symptomatic carriers. SARS-CoV-2 spreads primarily via respiratory droplets during close person-to-person contact in a closed space, especially a building. This article summarizes the environmental factors involved in SARS-CoV-2 transmission, including a strategy to prevent SARS-CoV-2 transmission in a building environment. SARS-CoV-2 can persist on surfaces of fomites for at least 3 days depending on the conditions. If SARS-CoV-2 is aerosolized intentionally, it is stable for at least several hours. SARS-CoV-2 is inactivated rapidly on surfaces with sunlight. Close-contact aerosol transmission through smaller aerosolized particles is likely to be combined with respiratory droplets and contact transmission in a confined, crowded, and poorly ventilated indoor environment, as suggested by some cluster cases. Although evidence of the effect of aerosol transmission is limited and uncertainty remains, adequate preventive measures to control indoor environmental quality are required, based on a precautionary approach, because COVID-19 has caused serious global damages to public health, community, and the social economy. The expert panel for COVID-19 in Japan has focused on the “3 Cs,” namely, “closed spaces with poor ventilation,” “crowded spaces with many people,” and “close contact.” In addition, the Ministry of Health, Labour and Welfare of Japan has been recommending adequate ventilation in all closed spaces in accordance with the existing standards of the Law for Maintenance of Sanitation in Buildings as one of the initial political actions to prevent the spread of COVID-19. However, specific standards for indoor environmental quality control have not been recommended and many scientific uncertainties remain regarding the infection dynamics and mode of SARS-CoV-2 transmission in closed indoor spaces. Further research and evaluation are required regarding the effect and role of indoor environmental quality control, especially ventilation.

## Background

In late December 2019, a cluster of severe pneumonia cases emerged in humans in Wuhan, Hubei Province, China [1, 2]. The causative pathogen was identified as a novel coronavirus that was named the severe acute respiratory syndrome coronavirus 2 (SARS-CoV-2) [3, 4]. The disease rapidly spread internationally, raising global public health concerns, and was subsequently termed coronavirus disease 19 (COVID-19) [5, 6]. The most common clinical manifestations of patients with COVID-19 are fever, cough, shortness of breath, and fatigue. Some patients have also shown radiographic ground-glass lung changes and eventually died of acute respiratory distress syndrome (ARDS) [7, 8]. The World Health Organization (WHO) declared COVID-19 as a global pandemic on March 11, 2020 [9].

SARS-CoV-2 is mainly transmitted human-to-human through close contact, respiratory droplets, fomites, and contaminated surfaces [10–13]. The WHO adapted a 1-m social distancing policy, based primarily on the assumption that the virus is transmitted through largely isolated droplets within this range [13]. However, the possibility of airborne transmission through airborne particles with diameters smaller than 5 μm has been suggested [14].

Several factors are involved in the transmission of SARS-CoV-2 between individuals, including the environment in buildings and human behavior [15–20]. Similar to the transmission routes of other respiratory viruses, such as influenza or human coronavirus [21–23], possible exposure pathways for COVID-19 infection by the SARS-CoV-2 are finger contact with virus-contaminated surfaces (fomites) and subsequent finger contact with the facial membranes; inhalation of the virus carried in airborne particles (inhalable or respirable particles) exhaled from cough or vocalization; and droplet spray, the direct projection of the virus carried in particles exhaled from cough or vocalization onto the facial membranes. Therefore, environmental factors in buildings, including temperature, humidity, stability on fomites, and ventilation and filtering systems, such as in public places, healthcare settings, restaurants, hotels, recreation facilities, or residential houses where people are close together, could have a significant influence on the infection. Adequate control of these environmental factors and proper human behavior in accordance with these environmental conditions play a significant role in preventing the spread of COVID-19 [24]. As most people spend more than 90% of their daily lives inside buildings, it is essential to understand the potential transmission dynamics of SARS-CoV-2 inside a building, the spatial dynamics, and the building operational factors that potentially promote and mitigate the transmission of SARS-CoV-2 and the spread of COVID-19.

This article aimed to review the effect of environmental factors in buildings, spatial dynamics, and building operational factors. In addition, a strategy to prevent SARS-CoV-2 transmission in building environments based on indoor environmental quality control recommended by the Japanese ministries is also summarized.

## Environmental factors involved in SARS-CoV-2 inside buildings

The characteristics of environmental air quality and environmental surfaces contaminated by the virus are important factors that determine the infectivity retention and extent and speed of the spread of the virus. The long-time persistence of these environmental factors influences the spread of COVID-19 [25]. The SARS-CoV-2 genetic material, ribonucleic acid (RNA), was detected in the rooms of both symptomatic and asymptomatic cruise ship passengers up to 17 days after the cabins were vacated [26]. The SARS-CoV-2 RNA was detected on the surfaces of the floor around the toilet, in the bedroom, bed pillow, phone, table, television remote control, chair arm, toilet flush button, toilet seat, and other items in the cruise ship [27]. Although the infectiousness of those materials is not known, the environment around the COVID-19 cases was contaminated extensively with SARS-CoV-2 during the COVID-19 outbreak on the cruise ship.

### Air temperature and humidity

The persistence of SARS-CoV-2 in aerosols in different environmental conditions has been reported. The results are summarized in Table 1. van Doremalen et al. compared the survival rate and half-life of SARS-CoV-2 and SARS-CoV-1 within 3 h of aerosolization at a temperature of 21 °C–23 °C and a relative humidity (RH) of 65%. Both viruses were detectable after 3 h of aerosolization and the median half-lives were 1.09 and 1.18 h for SARS-CoV-2 and SARS-CoV-1, respectively [28]. These results showed that the stability of SARS-CoV-2 was similar to that of SARS-CoV-1. Smither et al. reported that SARS-CoV-2 is more stable at a medium RH of 40–60% (decay rate, 0.91%/min) compared with a higher RH of 68–88% (decay rate, 1.59%/min) in tissue culture media (TCM), whereas the converse was observed in artificial saliva, with a decay rate of 2.27% per minute at a medium RH and 0.40% per minute at a higher RH [29]. The results of the half-life obtained with TCM at a medium RH were similar to those described above [28]. Although the infectious dose in humans is not known, SARS-CoV-2 may be able to remain viable and infectious in aerosols for hours (depending on the inoculum shed), if the virus is produced within small-particle aerosols.

**Table 1:** Persistence of coronaviruses in aerosols at different temperatures and relative humidity

Virus	Temperature (°C)	RH (%)	Virus load	Aerosolized time	Survival rate (%)	Decay rate (%/min)	Half-life (h)	Reference
SARS-CoV-2	21–23	65	10^3.5^ TCID_50_/L air	3 h	15.8	NR	1.09	van Doremalen et al. [28]
SARS-CoV-1	21–23	65	10^4.3^ TCID_50_/L air	3 h	15.8	NR	1.18	van Doremalen et al. [28]
SARS-CoV-2	19–22	40–60	10^6^ TCID_50_/mL aerosol of tissue culture media	90 min	NR	0.91	1.25	Smither et al. [29]
SARS-CoV-2	19–22	68–88	10^6^ TCID_50_/mL aerosol of tissue culture media	90 min	NR	1.59	NR	Smither et al. [29]
SARS-CoV-2	19–22	40–60	10^6^ TCID_50_/mL aerosol of artificial saliva	90 min	NR	2.27	0.5	Smither et al. [29]
SARS-CoV-2	19–22	68–88	10^6^ TCID_50_/mL aerosol of artificial saliva	90 min	NR	0.40	2.95	Smither et al. [29]

*Abbreviations*: *NR* not reported, *RH* relative humidity, *SARS-CoV* severe acute respiratory syndrome coronavirus, *TCID*_*50*_ tissue culture infectious dose 50

SARS-CoV-2 is susceptible to heat treatment but can persist for at least 14 days at refrigerated temperatures of 4 °C. With an increase in the incubation temperature, the time for virus inactivation was reduced dramatically to 2 days at 37 °C and to 5 min at 70 °C [30]. SARS-CoV-1 demonstrates tendencies similar to those of SARS-CoV-2 [31].

### Sunlight

In simulated saliva on a stainless steel surface, SARS-CoV-2 exhibits negligible decay over 60 min in darkness but loses 90% of its infectivity every 6.8–12.8 min, depending on the intensity of simulated ultraviolet (UV) B radiation levels, when exposed to simulated sunlight representative of the summer solstice at 40°N latitude at sea level on a clear day [32]. These data indicate that sunlight may inactivate SARS-CoV-2 rapidly on surfaces, suggesting that persistence and subsequently exposure risk may vary significantly between indoor and outdoor environments.

Experimental studies using SARS-CoV-2 aerosols produced from artificial saliva found that simulated sunlight inactivates the virus rapidly [33]. In dark conditions, the half-life of aerosolized SARS-CoV-2 is approximately 86 min in simulated saliva. The high-intensity sunlight-simulated summer was found to show a 90% reduction in infectious concentration after 6 min. Even with the low-intensity sunlight-simulated late winter or early fall, a 90% reduction was observed at 19 min. However, relative humidity (20, 37, 53, and 70%) had no significant effect on the survival of the aerosolized virus. These data indicate that sunlight is useful in mitigation strategies to minimize the potential for aerosol transmission.

### Stability on surfaces of fomites

SARS-CoV-2 can persist on plastic, stainless steel, and glass surfaces between 2 and 4 days at room temperature (Table 2) [28, 30]. The persistence of SARS-CoV-2 on those surfaces was similar to that of SARS-CoV-1 [28, 30, 31]. The survivability of these viruses on metal surfaces differed according to the type of metal. Both SARS-CoV-1 and SARS-CoV-2 survived for shorter periods on copper (8 and 4 h, respectively) than on stainless steel surfaces [28]. The antimicrobial properties of copper and copper alloy have been reported against various viruses [34, 35]. Several mechanisms have been proposed regarding copper-induced cellular toxicity to the virus. Reactive oxygen species generated by free copper ions cause the cells to commit metabolic suicide. Copper ions can induce protein destabilization on the virus. Copper ions have a direct effect on virus inactivation by causing aggregation of virus particles [35]. This might explain the short survival of SARS-CoV-1 and SARS-CoV-2 on copper surfaces compared with other metal surfaces. Studies have reported that copper alloys (≥58% copper) reduced the surface microorganisms when incorporated into various hospital furnishings and fittings [36, 37]. The use of copper in combination with optimal infection-prevention strategies may further reduce the risk of patients and healthcare workers acquiring COVID-19 infection in healthcare environments.

**Table 2:** Persistence of coronaviruses on surfaces and fomites

	Fomite	Virus	Temperature (°C)	RH (%)	Persistence	Time of complete decay	Half-life (h)	Reference
Non-porous surfaces	Plastic	SARS-CoV-2	NR	NR	3 days	4 days	6.8	van Doremalen et al. [28]
	Plastic	SARS-CoV-2	22	65	4 days	7 days	11.4	Chin et al. [30]
	Plastic	SARS-CoV-1	NR	NR	3 days	4 days	7.6	van Doremalen et al. [28]
	Plastic	SARS-CoV-1	21–25	NR	4 days	5 days	NR	Duan et al. [31]
	Copper	SARS-CoV-2	NR	NR	4 h	8 h	0.8	van Doremalen et al. [28]
	Copper	SARS-CoV-1	NR	NR	8 h	1 day	1.5	van Doremalen et al. [28]
	Stainless steel	SARS-CoV-2	NR	NR	3 days	4 days	5.6	van Doremalen et al. [28]
	Stainless steel	SARS-CoV-2	22	65	4 days	7 days	14.7	Chin et al. [30]
	Stainless steel	SARS-CoV-1	NR	NR	2 days	3 days	4.2	van Doremalen et al. [28]
	Glass	SARS-CoV-2	22	65	2 days	4 days	4.8	Chin et al. [30]
	Glass	SARS-CoV-1	21–25	NR	4 days	5 days	NR	Duan et al. [31]
Porous surfaces	Cloth	SARS-CoV-2	22	65	1 day	2 days	NR	Chin et al. [30]
	Cloth	SARS-CoV-1	21–25	NR	5 days	>5 days	NR	Duan et al. [31]
	Surgical mask–outer layer	SARS-CoV-2	22	65	7 days	>7 days	23.9	Chin et al. [30]
	Surgical mask–inner layer	SARS-CoV-2	22	65	4 days	7 days	9.9	Chin et al. [30]
	Paper	SARS-CoV-2	22	65	30 min	3 h	NR	Chin et al. [30]
	Tissue paper	SARS-CoV-2	22	65	30 min	3 h	NR	Chin et al. [30]
	Banknote paper	SARS-CoV-2	22	65	2 days	4 days	7.9	Chin et al. [30]
	Press paper	SARS-CoV-1	21–25	NR	4 days	5 days	NR	Duan et al. [31]
	Filter paper	SARS-CoV-1	21–25	NR	5 days	>5 days	NR	Duan et al. [31]
	Cardboard	SARS-CoV-2	NR	NR	1 day	2 days	3.5	van Doremalen et al. [28]
	Cardboard	SARS-CoV-1	NR	NR	8 h	1 day	0.6	van Doremalen et al. [28]
	Wood	SARS-CoV-2	22	65	1 day	2 days	NR	Chin et al. [30]
	Wood boards	SARS-CoV-1	21–25	NR	4 days	5 days	NR	Duan et al. [31]

*Abbreviations*: *NR* not reported, *RH* relative humidity, *SARS-CoV* severe acute respiratory syndrome coronavirus

SARS-CoV-2 showed variable persistence on different porous surfaces, such as paper, cardboard, wood, cloth, and mask. SARS-CoV-2 survived on the inner and outer layers of surgical facemasks for 4 and 7 days, respectively [30]. In other words, the infectious virus can be recovered from a surgical mask after 7 days (22 °C, 65% RH). SARS-CoV-2 survived for 1 day on cardboard, wood, and cloth [28, 30]. It survived for 2 days on banknote paper [30]. However, the virus survived for only 30 min on paper and tissue paper, with complete decay after 3 h [30]. Under the same conditions, SARS-CoV-2 survived for a longer time (1 day) than SARS-CoV-1, which survived for only 8 h [28].

Interestingly, the stability of SARS-CoV-2 was enhanced when present with bovine serum albumin, which is used commonly to represent sources of protein found in human sputum [38]. Conversely, SARS-CoV-2 decayed more rapidly when either the humidity or the temperature was increased, but the droplet volume and surface type (stainless steel, plastic, or nitrile glove) did not impact the decay rate significantly. At room temperature (24 °C), the virus half-life ranged from 6.3 to 18.6 h depending on the relative humidity but was reduced to 1.0–8.9 h when the temperature was increased to 35 °C [39].

These findings suggest that a potential for fomite transmission may persist for hours to days in indoor environments and that the survivability of fomites is affected by temperature and relative humidity, as well as by the presence of protein found in human sputum.

In summary, virus survival decreases with an increase in temperature. Maintaining temperatures above 60 °C for more than 60 min usually inactivate most viruses [40]. SARS-CoV-2 is also susceptible to heat treatment. Persistence of SARS-CoV-2 on dry inanimate surfaces was range for 1–7 days. The persistence of influenza virus, rhinovirus, and norovirus was reported at 1–2 days, 2 h −7 days, and 8 h −7 days, respectively [41]. Thus, SARS-CoV-2 can remain infectious on the surfaces compared with the influenza virus. However, the persistence of SARS-CoV-2 was significantly low on copper as compared with other surfaces such as plastics, stainless steel, glass, and fabrics. In addition, sunlight can rapidly inactivate SARS-CoV-2. These findings might be helpful in designing methods to significantly decrease viral transmission inside buildings.

## Possible pathways of SARS-CoV-2 transmission

Droplet and contact transmission have accounted for the main routes of infection related to COVID-19. In addition, the WHO announced the possibility of aerosol infection in specific circumstances, such as endotracheal intubation, bronchoscopy, etc. [42]. On the other hand, some researchers have claimed a risk of short- and medium-range person-to-person distance in aerosol transmission, but evidently, airborne transmission (long-range distance) has not been acknowledged [43].

Nicas et al. estimated that the relative contributions of four influenza virus exposure pathways, namely (1) virus-contaminated hand contact with facial mucous membranes, (2) inhalation of respirable cough particles, (3) inhalation of inspirable cough particles, and (4) the spray of cough droplets onto facial mucosa, account for 31, 17, 0.52, and 52% risk of influenza infection, respectively [22]. The transmission routes for these cough particles can be categorized as “droplet transmission,” where droplets (>5 μm diameter, traveling <1 m) containing viable viruses make contact with the nose, mouth, eyes, or upper respiratory tract; and “airborne transmission” where droplet nuclei (<5 μm diameter, which can travel >1 m) are inhaled by susceptible individuals [44].

The WHO has stated that airborne transmission is different from droplet transmission as it refers to the presence of microbes within droplet nuclei, which are generally considered to be particles smaller than 5 μm in diameter, can remain in the air for long periods of time and can be transmitted to others over distances greater than 1 m [42]. From the view of this statement, the COVID-19 virus can be transmitted by direct contact via infected people and indirect contact via surfaces in the immediate environment or with objects used by the infected person, not by airborne transmission through small airborne particles.

Recently, 36 researchers insisted on the potential risk of indoor airborne transmission of SARS-CoV-2 and the importance of sufficient and effective ventilation, particle filtration, and air sterilization as infection control measures inside buildings [43]. The WHO recognized the potential risks of the airborne spread of COVID-19 [13]. However, a recent experimental study using transgenic mice indicated that SARS-CoV-2 could be experimentally transmitted among mice by close contact, through respiratory droplets, but is hardly transmitted through exposure to airborne particles [45]. A recent study also indicated that the role of airborne particles as carriers of the virus diffusion is not evident [46]. In discussing the airborne transmission of SARS-CoV-2, it is important to understand the characteristics of aerosol particles emitted by cough and speech in indoor environments. This section describes briefly the size distribution of droplets, the emission rate of droplets by cough and speech, and the resuspension of particles deposited on floor surfaces.

SARS-CoV-2 has a diameter of 60–140 nm, with characteristic spikes ranging from 9 to 12 nm [47]. Johnson et al. determined an aerosol droplet size distribution ranging from 700 nm to 1 mm, generated by breathing, speech, and voluntary coughing by using the expired droplet investigation system with an aerodynamic particle sizer (APS) and a droplet deposition analysis (DDA) [48]. In the case of speech, three different droplet size distribution modes were identified, with median diameters at 1.6, 2.5, and 145 μm. In the case of voluntary coughing, the modes were located at 1.6, 1.7, and 123 μm. Therefore, a wide range of droplet sizes is emitted by speaking and coughing. The key point in this study was that small droplets can be emitted not only by speaking and coughing but also through breathing. Individuals infected by SARS-CoV-2 without symptoms can also transmit the infection to others. The emission by natural breathing without coughing or sneezing is also important to understand the transmission from infected individuals without symptoms.

Asadi et al. placed APS in a laminar flow hood to characterize the number and size distribution of particles emitted by individual human volunteers while performing various vocalizations and breathing activities [49]. The results showed that the rate of particle emission during normal human speech is correlated positively with the loudness (amplitude) of vocalization, ranging from approximately 1 to 50 particles per second (0.06 to 3 particles/cm^3^) for low to high wavelengths, regardless of the language spoken (English, Spanish, Mandarin, or Arabic). The results also indicated that the droplets could be emitted not only by speech but also by singing a song, cheering loudly for sports games, and so on.

Rahmani et al. reviewed the methods for the sampling and detection of coronaviruses, especially SARS-CoV-2. Most of the samplers used, such as polytetrafluoroethylene filters, gelatin filters, and cyclones, showed a suitable performance to trap SARS-CoV and Middle East respiratory syndrome (MERS)-CoV, followed by polymerase chain reaction (PCR) analysis [50]. Some studies reported detecting SARS-CoV-2 from patients’ rooms in hospitals, although it seems difficult to discriminate whether these were airborne or transmitted through respiratory droplets because sampling conditions (i.e., the patient’s distance from the sampler, patient’s activities, coughing and sneezing during sampling time, etc.) can affect the results.

Liu et al. investigated airborne SARS-CoV-2 by measuring viral RNA in aerosols in two different hospitals in Wuhan during the COVID-19 outbreak in February and March 2020 [16]. The unique point in this study was to investigate the size distributions of airborne SARS-CoV-2 droplets. Aerosol samples were collected using a miniature cascade impactor (Sioutas Impactor, SKC) that could separate aerosols into five ranges (>2.5, 1.0–2.5, 0.50–1.0, and 0.25–0.50 μm on 25-mm filter substrates and 0–0.25 μm on 37-mm filters) and determined through the quantification of their genetic material (RNA). SARS-CoV-2 aerosols were found to include mainly two size ranges, one in the submicrometer region (d_p_ 0.25–1.0 μm) and the other in the supermicrometer region (d_p_ > 2.5 μm), in isolation wards of ventilated patient rooms and medical staff areas. The authors hypothesized that the source of the submicrometer virus-laden aerosols may be resuspension from the surface of the protective apparel worn by medical staff while they are removing the equipment.

The resuspension occurs largely because of the particle surface properties, human activities, environmental conditions, and so on. Resuspension occurs mainly as a result of human activities, especially walking in indoor spaces. Qian et al. reviewed particle resuspension owing to human walking in indoor environments [51]. From the results presented, resuspension is an important source compared with other indoor sources, such as cooking and stoves, and resuspension increases with particle size in the range of 0.7–10 μm. Many researchers proposed resuspension terms, such as a resuspension rate coefficient (h^−1^), a resuspension fraction (-), an emission rate (mg/h), a resuspension factor (m^−3^), and a resuspension emission factor (mg/mg). Rosati et al. experimentally investigated the characterization of the size distribution of resuspended particle matter from the carpet during walking events [52]. The resuspension emission factor by particle size was defined as the resuspension emission rate by particle load on the carpet by particle size. The emission factors were approximately 10^−4^ to 10^−1^ of particle diameter 1 to 10 μm. Therefore, a small portion of particles deposited on the floor can be resuspended in indoor air. The resuspension is progressively more likely to occur by larger particles than by smaller ones. Although there are a few studies on bioaerosols, and especially virus particles, bioaerosol resuspension is clearly not the same as infectious virus resuspension.

It is important for airborne transmission to understand the droplet diameter emitted from patients. Previous studies indicated that an aerosol droplet size distribution was ranged from 700 nm to 1 mm, generated not only by speaking and coughing but also through breathing. From the field measurements in the hospital, SARS-CoV-2 aerosols could be detected to include mainly two size ranges, 0.25–1.0 μm and larger than 2.5 μm, in isolation wards of ventilated patient rooms and medical staff areas. Moreover, the resuspension is progressively more likely to occur by larger particles than by smaller ones, but bioaerosol resuspension cannot be clearly the same as infectious virus resuspension.

## Cases of SARS-CoV-2 transmission owing to environmental factors

The number of secondary infections of COVID-19 varies widely, and most outbreaks of many secondary infections occur under common indoor environmental factors [53, 54]. Given the lack of data to define environmental control measures against COVID-19 based on quantitative analysis, it is important to understand the conditions common to outbreak cases and to take measures to minimize the indoor environmental factors that promote infection. In this section, we summarize the cases of outbreaks in which indoor environmental factors, including human behavior, are believed to have facilitated infection, as well as the environmental survey of SARS-CoV-2.

### Restaurant in Guangzhou, China

On January 24, 2020, a COVID-19 outbreak occurred in a restaurant in Guangzhou, China, infecting ten people in three families. The minimum length of time during which the index-infected person was present with the secondary infected person was 46 min, and the possibility of contact infection was considered to be low based on in-store camera recordings. Fifty percent (five of ten) of those at the same table as the infected person were found to be infected within the next 7 days. At the adjacent leeward table, 75% (three of four) were infected. Two of the seven people at the windward table were infected. Other diners who were located away from the airflow around the infected tables, as well as the staff, were not infected. Lu et al. concluded that the airflow from the air-conditioners promoted droplet infection and recommended that distances between people are maintained and ventilation improved [55].

In this case, Li et al. conducted a detailed investigation of the indoor environment by measuring the ventilation rate and conducting a numerical thermo-fluid analysis. As a result, they found that only the ventilation fan in the restroom was in operation. The ventilation fan on the wall of the restaurant was sealed and not in operation, and the ventilation rate ranged from 0.56 to 0.77 air changes per hour (ACH) and 0.75 to 1.04 L/s per person. They suggested that poor ventilation may have been the primary cause of the spread of the infection by aerosol transmission [56].

### Call center in Seoul, South Korea

Ninety-four people tested positive for the virus on one floor of the call center, where 216 employees worked. The infection is believed to have occurred over a 16-day period, beginning on February 21. Of the 94 people infected, 92 developed the disease and two were asymptomatic. The fact that the infection was concentrated on one side of the office, while the number of infected individuals on the other side was very low suggests that SARS-CoV-2 can spread easily in a crowded workplace environment, such as a call center. It also points out that although there may have been many contacts with workers on different floors in elevators, lobbies, etc., the infection was confined mostly to a single floor. This indicates that contact time may be a major factor in promoting the spread of SARS-CoV-2 [57].

### Washington State Squadron practice, United States of America

Of 61 people who participated in a choir practice on March 10, 2020, 53 cases were identified, including 33 confirmed cases and 20 possible cases, among those experiencing at least one symptom of COVID-19. The secondary infection rate was 53.3% from confirmed cases and 86.7% from all cases. Three of the 53 patients were hospitalized (5.7%) and two died (3.7%). During the 2.5-h singing practice, members sat close to each other. At the end of the practice, they piled up chairs, thus increasing the chance of infection by aerosols or contact. The act of singing itself may have contributed to the infection by the release of aerosols, related to the loudness of the voice [49, 58].

### Meat-processing plant in North Rhine-Westphalia, Germany

Meat-processing plants have emerged as hotspots for SARS-CoV-2 around the world. An outbreak of SARS-CoV-2 at Germany’s largest meat-processing facility resulted in more than 1400 confirmed infections during tests conducted a month after the initial outbreak. Guenther et al. conducted a causal investigation into the May 2020 outbreak. It was suggested that the infected person, who was the first example of a supplemental case, transmitted the virus to a colleague more than 8 m away over 3 consecutive working days. The low temperature in an environment with the low intake of outside air and air circulation through the air-conditioning system in the hall, combined with the high physical workload of workers with heavy breathing, have been suggested as factors that enabled transmission over distances greater than 8 m by virus-containing aerosol particles. They stated that under these circumstances, distances of 1.5–3 m are not sufficient to prevent infection and that the wearing of masks, improved ventilation and airflow, and the installation of filtering devices are necessary to reduce the risk of infection [59].

These outbreaks strongly suggest that in a confined, crowded, and poorly ventilated environment where conversations, loud vocalizations, and heavy breathing take place, SARS-CoV-2 can spread through the air at a distance of 2 m or more and may result in a large number of secondary infections.

## Control of infectious aerosols by ventilation, filtration, and ultraviolet germicidal irradiation

### Possibility of controlling aerosols by ventilation

The WHO defines droplets and droplet nuclei as respiratory aerosols more than 5 μm in diameter and the residue of dried respiratory aerosols up to 5 μm in diameter, produced by the evaporation of droplets coughed or sneezed into the atmosphere or aerosolized infective material, respectively [60]. Based on field measurement results at Wuhan hospitals during the COVID-19 outbreak, Liu et al. reported that the peak concentration of SARS-CoV-2 aerosols appears in two distinct size ranges: at the submicron scale with dominant aerodynamic diameters between 0.25 and 1.0 μm; and at the supermicron scale with diameters greater than 2.5 μm [16]. The main sources of SARS-CoV-2 aerosols are coughs and sneezes by infected persons. The capacity for droplets to travel long distances in airflow is determined largely by their size. Most communicable respiratory infections are transmitted via large droplets over short distances or contact with contaminated surfaces. Large droplets (diameter, >60 μm) tend to settle quickly from the air, and thus the risk of pathogen transmission is limited to individuals in close proximity to the saliva droplet source. Small droplets (diameter, ≤60 μm) may be involved in short-range transmission (i.e., when the distance between individuals is less than 1 m) and are likely to evaporate into droplet nuclei (diameter, <10 μm) in favorable environments, making them candidates for long-distance aerosol transmission [61].

The terminal settling velocity of a particle increases rapidly with its size, as it is proportional to the square of particle diameter. The terminal settling velocity of a particle up to 10 μm in size is less than 0.3 cm/s (0.003 m/s) [62], and because the particle diameter decreases as a result of the evaporation of the droplets during settling, droplets will remain suspended for a longer time indoors based on the relative indoor humidity [63]. Therefore, aerosols up to 10 μm in diameter are carried easily over long distances (final inlet air) by the indoor airflow generated by air conditioning or ventilation equipment. Field measurements show that the largest and average velocities in occupant space are 0.4 and 0.1 m/s, respectively [64, 65]. It is possible to control aerosols containing a virus with a proper indoor airflow plan.

### Relationship between the air change rate and the probability of infection

Regarding the indoor airflow plan, there are different types of ventilation systems such as piston flow ventilation and mixing-type ventilation. When the infected individual’s position can be fixed, piston flow ventilation or push-pull ventilation is applicable. However, as the positions of infected people cannot be pinpointed in general environments, mixing-type ventilation is more effective because it dilutes the infectious aerosols to decrease their concentration in the air.

To describe the relationship between the air exchange rate and the probability of infection, the Wells–Riley model was used:

1 $$ {P}_I=\frac{C}{S}=1-{e}^{-\frac{Iqpt}{Q}} $$

where

*PI* = the probability of infection (–),

*C* = the number of susceptible individuals to become infected (–),

*S* = the number of susceptible individuals (–),

*I* = the number of infectious individuals (–),

*p* = the pulmonary ventilation rate of a person (m^3^/h),

*q* = the generation rate of infectious quanta (h^−1^),

*t* = the exposure time (h),

and *Q* = the room ventilation rate with clean air (m^3^/h).

This model is based on the assumption that the air in the room is well mixed, leading to a uniform concentration of bioaerosols throughout the space. To estimate the probability of infection in a room, the quantum generation rate must be determined. A quantum represents the minimum dose that can cause infection in the host [66], and the quantum generation rate is the number of quanta produced per hour per infectious individual. Table 3 provides the values of quantum generation rates reported for different infectious aerosols [77]. Dai and Zhao estimated the quantum generation rate of COVID-19 by fitting the relationships between known rates and R0 (the basic reproduction number) of the infectious agents listed in Table 3 [78]. The results indicate that the quantum generation rate for COVID-19 ranges from 14 to 48 h^−1^.

**Table 3:** Reported quantum generation rates for several infectious aerosols

	Quanta (h^−1^)	Commonly used value	References
Influenza	~15 to ~500	67 or 100	Rudnick and Milton [67]; Liao et al. [68]; Beggs et al. [69]; Sze To and Chao [70]
Rhinovirus	~1 to ~10		Rudnick and Milton [67]
Tuberculosis	~1 to ~50	~13	Nardell et al. [71]; Escombe et al. [72]; Beggs et al. [69]; Chen et al. [73]
SARS-CoV-1	~1 to ~300		Liao et al. [68]; Qian et al. [74]
Measles	~570 to ~5,600	5480	Riley et al. [75]; Riley et al. [76]

*Abbreviation*: *SARS-CoV* severe acute respiratory syndrome coronavirus

**Fig. 1:**
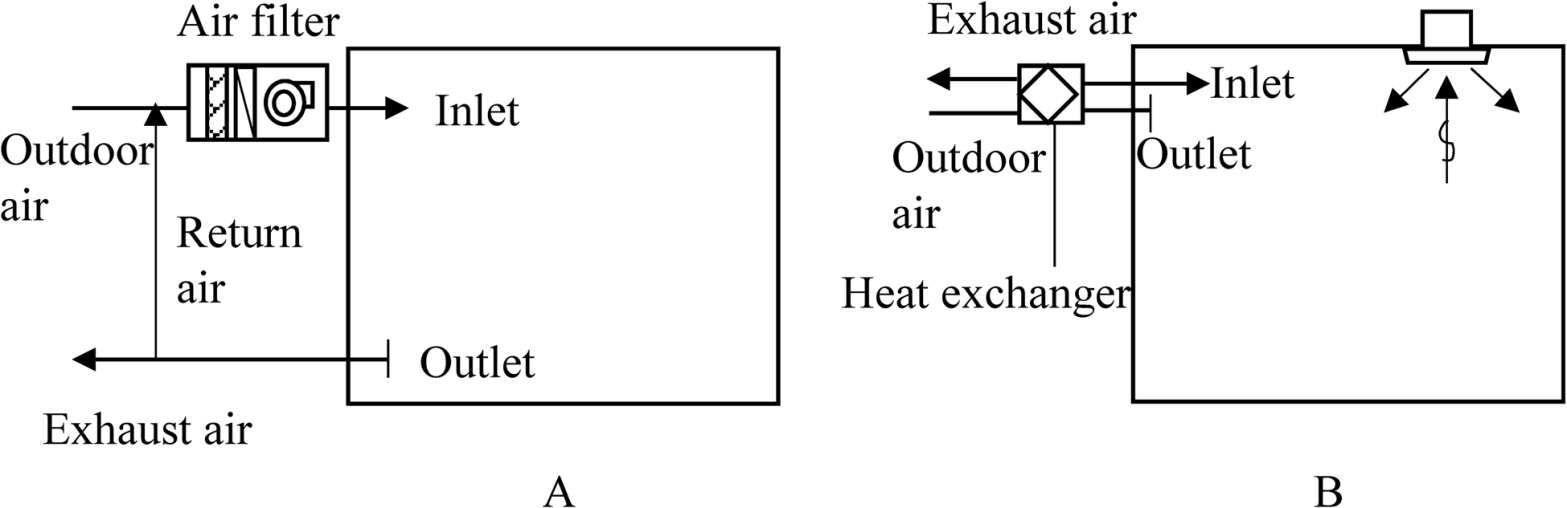
Traditional Japanese office building HVAC systems: **a** a centralized HVAC system; and **b** a centralized ventilation system with an individual air-conditioning system

Figure 1 shows the schematics of the two typical heating, ventilation, and air-conditioning (HVAC) systems used in Japanese office buildings. System A is the centralized HVAC system (CS) and system B is the individual HVAC system (IS). The ventilation rates of the CS are generally approximately 2 ACH for outdoor air and 4 ACH for return air. Moreover, the CS normally uses an air filter with a collection efficiency approximately equivalent to a minimum efficiency reporting value (MERV) of 12, indicating a removal efficiency of 90% of droplets from human respiration activities, most of which are less than 5–10 μm in diameter [79–81]. In this case, the equivalent change rate is approximately 5.6 ACH (=2 ACH + 4 ACH × 90%).

**Fig. 2:**
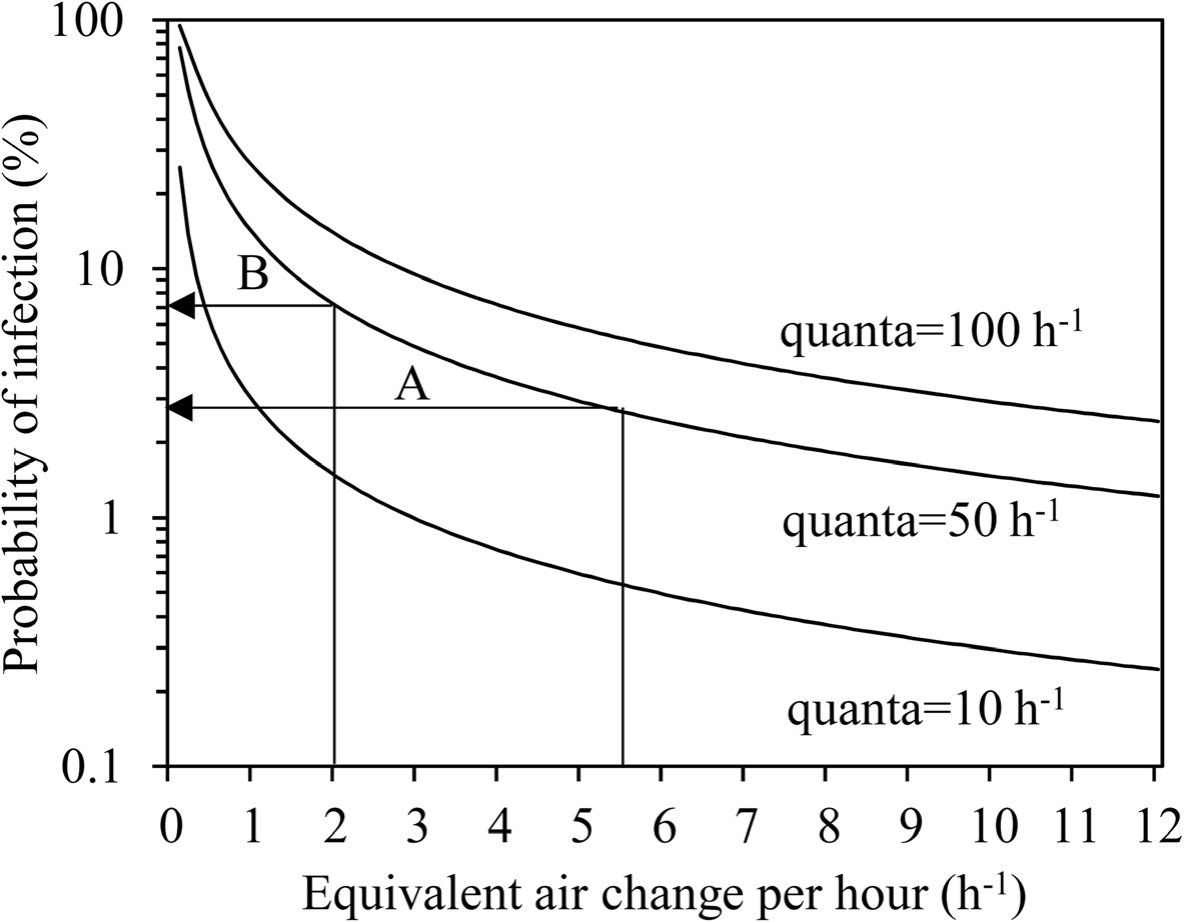
Probability of infection plotted against the equivalent air changes per hour. Conditions: *I* = 1 person; *p* = 0.48 m^3^/h; *t* = 8 h; floor area = 500 m^2^; room volume = 1300 m^3^

Figure 2 shows the probability of infection plotted against the equivalent air change rate (hourly rate of room ventilation with clean air) based on Eq. (1). The prediction conditions are shown as *I* = 1 person, *p* = 0.48 m^3^/(hour⋅person), *t* = 8 h, room floor area = 500 m^2^, ceiling height = 2.6 m. The higher the equivalent air change rate (the room ventilation rate with clean air/room volume), the lower the probability of infection. Furthermore, the CS shows a lower probability of infections than the IS because of the larger amount of clean air.

### Filtration

The use of highly efficient particle filtration in centralized HVAC systems reduces the airborne load of infectious particles [82, 83]. The collection efficiency of air filters for suspended particles by particle size is given in Table 4. In Japanese office buildings, medium-efficiency air filters (MERV 11–13 in Table 4) are typically used, whereas high-efficiency particulate air filters (HEPA: 99.97% or higher particle collection efficiency for particles sized 0.3 μm at the rated airflow volume) are used for rooms such as hospital operating rooms that demand high air cleanliness.

**Table 4:** Minimum efficiency reporting values and filter efficiencies by particle size in ASHRAE Standard 52.2-2017

MERV	0.3–1.0 μm	1.0–3.0 μm	3.0–10 μm
1	n/a	n/a	E_3_ < 20
2	n/a	n/a	E_3_ < 20
3	n/a	n/a	E_3_ < 20
4	n/a	n/a	E_3_ < 20
5	n/a	n/a	20 ≤ E_3_
6	n/a	n/a	35 ≤ E_3_
7	n/a	n/a	50 ≤ E_3_
8	n/a	20 ≤ E_2_	70 ≤ E_3_
9	n/a	35 ≤ E_2_	75 ≤ E_3_
10	n/a	50 ≤ E_2_	80 ≤ E_3_
11	20 ≤ E_1_	65 ≤ E_2_	85 ≤ E_3_
12	35 ≤ E_1_	80 ≤ E_2_	90 ≤ E_3_
13	50 ≤ E_1_	85 ≤ E_2_	90 ≤ E_3_
14	75 ≤ E_1_	90 ≤ E_2_	95 ≤ E_3_
15	85 ≤ E_1_	90 ≤ E_2_	95 ≤ E_3_
16	95 ≤ E_1_	95 ≤ E_2_	95 ≤ E_3_

*Abbreviations*: *MERV* minimum efficiency reporting value, *n/a* not available

### Ultraviolet germicidal irradiation

It is known that ultraviolet germicidal irradiation (UVGI) systems are used for air and surface disinfection. Investigations of the bactericidal effect of sunlight in the late 19th century planted the seed of air disinfection by UV radiation. The first to nurture this seed was Wells, who both discovered the spread of airborne infection by droplet nuclei and demonstrated the ability of UVGI to prevent such a spread. With modern concerns regarding multi- and extensive drug-resistant tuberculosis, bioterrorism, influenza pandemics, and severe acute respiratory syndrome, interest in UVGI continues to grow [84]. The Centers for Disease Control and Prevention (CDC) [85] and the American Society of Heating, Refrigerating and Air-Conditioning Engineers (ASHRAE) [86] recommend the application of UVGI to fight airborne diseases.

Microbes are uniquely vulnerable to light of wavelengths at or near 253.7 nm because the maximum absorption wavelength of a deoxyribonucleic acid (DNA) molecule is 260 nm. The chemical compound pyrimidine in a DNA base strongly absorbs UV light. After irradiation, the DNA sequence where two pyrimidines link can form pyrimidine dimers. These dimers can change the DNA double-helix structure and interfere with DNA duplication, as well as lead to the destruction of the replication ability of cells and thus render the cells noninfectious [87, 88].

Regarding the sterilization or inactive performance of UVC, the population St of microbes exposed to any biocidal factor is described by the characteristic logarithmic decay equation:

2 $$ {S}_t={e}^{- kIt} $$

where

*k* = the standard decay-rate constant (cm^2^/mW⋅s),

*I* = the intensity of UVGI (mW/cm^2^),

and *t* = the exposure time (s).

The standard decay-rate constant of the influenza A virus has been reported as 0.001187 cm^2^/μW⋅s [89–91].

Recently, upper-room UVGI and in-duct UVGI have been the two primary applications of UVGI air disinfection. In-duct UVGI is designed to disinfect air as it passes through the HVAC system before it is recirculated or exhausted, and it has been also suggested to reduce nonspecific building-related illnesses [92, 93].

In summary, since small droplets (diameter, ≤60 μm) are likely to evaporate into droplet nuclei (diameter, <10 μm) in favorable environments, and the terminal settling velocity of a particle up to 10 μm in size is lower than 0.3 cm/s (0.003 m/s), it is possible to control aerosols containing a virus with a proper indoor airflow plan and sufficient ventilation rates. Moreover, filtration is effective in the reduction of aerosol concentration. Ventilation and filtration can contribute to the reduction of indoor infection probability. On the other hand, it is well-known that UVGI is an effective measure against microbes. The WHO, the CDC, and the ASHRAE recommend the application of UVGI to fight airborne diseases. The application in Japan will be expected.

## Indoor environmental quality control as a strategy to prevent SARS-CoV-2 transmission in a building environment

It is important to decide the adequate measures against a new infectious agent by analyzing actual infection cases and to execute them as soon as possible. As of February 26, 2020, the Ministry of Health, Labour and Welfare (MHLW) COVID-19 Response Team examined a total of 110 cases among 11 clusters and investigated who acquired infection from whom. All clusters were associated with close contact in indoor environments, including fitness gyms, a restaurant boat on a river, a club with live music, healthcare facilities, and a snow festival where there were eating spaces in tents with minimal ventilation rate [54]. Therefore, the MHLW published a document titled “Prevention of the COVID-19 Clusters” on March 1, 2020 [94], showing the need for adequate ventilation in buildings because a ventilation standard for infection control has not been established in general buildings in Japan and the characteristics of indoor spaces where the clusters occurred might include poor ventilation and crowding. Therefore, the “3 Cs,” namely, “closed spaces with poor ventilation,” “crowded spaces with many people,” and “close contact,” such as from intimate conversations, loud cheering, singing, or exercise within a short distance from other individuals, were proposed as important factors that result in a COVID-19 cluster [95, 96].

The MHLW published “Ventilation to Improve “Closed Spaces with Poor Ventilation” in Commercial Facilities” on March 30, 2020, based on the analysis by the MHLW COVID-19 Response Team concerning the actual ventilation state against the standards in the Law for Maintenance of Sanitation in Buildings, and recommended adequate ventilation rates in accordance with the standards of the law and the increase in the ventilation rates by adjusting the ventilation systems and opening the windows [97]. Furthermore, this recommendation was added in the “Measures to Prevent the Large- Scale Spread of COVID-19 in Workplaces” on March 31, 2020 [98]. On April 2, 2020, a document titled “Maintenance of Air-Conditioning and Ventilation Systems in Specific Buildings” was published and adequate ventilation was requested again [99]. Moreover, on April 3, 2020, the managers of commercial facilities were informed of the “Methods of Ventilation for Improving “Closed Spaces with Poor Ventilation,” and the guidelines of ventilation measures in both specific buildings and other buildings were provided [100]. In these documents, the required ventilation rate of approximately 30 m^3^/h per person was recommended [94]. When this ventilation rate was determined, the results of studies on tuberculosis [101–103] and measles [104], which are infectious diseases known to be spread by airborne transmission, were considered [97]. The ventilation rates needed are indicated not per room but per person and if adequate ventilation is not secured in the room, the number of persons should be limited to avoid overcrowding, which may be a factor in clusters of infected individuals. The ventilation rates needed to prevent SARS-CoV-2 transmission have not been reported; thus, opening windows has also been recommended. However, it was difficult to recommend the specific methods because the conditions of the rooms (e.g., the number of windows, and the width and direction of the windows) are different.

In the expert meeting on Novel Coronavirus Disease Control on May 4, a “new lifestyle” for the long-term fight against COVID-19 was suggested, comprising three basic measures: social distancing, wearing face masks, and washing hands [105]. Since the middle of April, researchers on building hygiene have collected recent scientific findings and discussed the ventilation measures towards summer [62]. During the rainy season and summer, opening the windows may create a poor indoor environment with insufficient air-conditioner cooling and dehumidification, resulting in increased risks of heatstroke, insomnia, and allergies owing to mold and mites. After discussions, the researchers produced a document titled “Indoor Environmental Measures in Summer Against COVID-19: the Suggestion From the Researchers on Building Hygiene” on May 20, 2020 and provided it to the expert meeting members on Novel Coronavirus Disease Control and headquarters of the MHLW [62]. From the present evidence, it is difficult to indicate specific standard values, such as ventilation rates. Thus, ventilation and air-conditioning measures recommended under some conditions were shown, see Table 5.

**Table 5:** Recommendations on the ventilation and air-conditioning measures to prevent COVID-19 infection

Spaces	Recommendation
Every indoor space	• Enough ventilation is necessary to prevent COVID-19 infection.
	• Opening windows is effective for ventilation and opening them wide and for a longer time is desirable.
	• In summer, air conditioners are necessary to prevent health risks such as heatstroke, etc.
	• General air conditioners do not function as ventilators, so mechanical ventilation and opening windows are necessary.
	• When opening windows, it is necessary to prevent harmful insects and animals from coming in.
Spaces in which air conditioning and ventilation systems are installed	• It is necessary to check the systems and to confirm enough ventilation rates are secured.
	• It is necessary to limit the number of persons in a room or shorten the time they are inside secure enough ventilation rates per person.
	• It is necessary to investigate what the building is used for, how often it is used, what kind of air-conditioning and ventilation system is used in the building when taking such measures as the better control of air-conditioning and ventilation systems, or the use of air cleaners, and the use of humidifiers in winter.

*Abbreviation*: *COVID-19* coronavirus disease 2019

On May 26, 2020, the Ministry of the Environment and the MHLW served local governments a notice of “Action for Prevention of Heatstroke in 2020,” which requested that they take action to prevent heatstroke under the spread of COVID-19 [106]. The headquarters of COVID-19 prevention in the MHLW considered expert opinions, scientific literature, international standards, and laws and regulations in Japan in the search for effective methods, and published a document titled “Ventilation for Improving “Closed Spaces with Poor Ventilation” with Prevention of Heatstroke” on June 17, 2020 [107]. In addition, it published the document titled “Methods of Ventilation for Improving “Closed Spaces with Poor Ventilation” with Prevention of Heatstroke for Owners Using Air-Conditioning System without Ventilation in Commercial Facilities” on June 24, 2020 [108]. The document shows points to remember while opening windows for ventilation, in using air cleaners, and so on. Furthermore, in the “Q&A for the Public,” information was provided on home air conditioners and ventilation during the hottest season. It was indicated that air conditioners should be used to prevent heatstroke, but that general air conditioners do not have a ventilatory function; thus, the whole air of a room should be ventilated twice or more per hour and the use of a 24-h mechanical ventilation system is effective.

The Society of Heating, Air-conditioning and Sanitary Engineering of Japan started a special committee against COVID-19 and published a document titled “Is COVID-19 Spread by Air-Conditioning or Ventilation?—Experts’ Opinions” on June 16, 2020. In the document, the relationship between air-conditioning or ventilation and the risk of infection, the possibility of the spread of COVID-19 through air conditioners, the infection risk by inadequate ventilation and filters, and the prevention of heatstroke with infection control are discussed and the following remarks were obtained [109]:

- In Japanese buildings, which are designed and managed in accordance with the Law for Maintenance of Sanitation in Buildings, considering the performance of ventilation and filters, the risk of the spread of COVID-19 in a room through air-conditioning systems is believed to be very low. However, in the case of buildings without the general-performance filters in air conditioners, commercial package-type air conditioners, or a fan coil unit (i.e., FCU), another ventilation method should be added.
- Securing ventilation rates per person may prevent droplet infection through airflow. In hospitals and clinics where individuals can wear face masks all the time, the droplet from infected individuals can be prevented.
- When adequate ventilation rates are secured, it is necessary to use air conditioners to prevent heatstroke in summer. In hospitals and clinics where individuals are believed to be infected, the airflow from air conditioners should not be directed to any person.
- Considering the fact that in a poorly ventilated room, an individual was infected when spending time with an infected person for less than an hour, not intermittent but continuous ventilation is desirable.
- In cases where natural ventilation is available, it is desirable to secure ventilation rates by opening the windows and so on if an adequate room temperature is secured. However, in the buildings designed and managed in accordance with the Law for Maintenance of Sanitation in Buildings, natural ventilation may cause a bad influence on air balance and may be beyond the standards of the law. Thus, it is desirable to consult experts.

An industry-classified guideline for the prevention of the spread of COVID-19 was made based on the proposal by the experts’ meeting on May 4, 2020. As of writing this article, approximately 100 guidelines were available on the website of the Cabinet Secretariat of the Japanese government [110]. The items concerning building environments are cleaning, disinfection, ventilation, room density, etc., and guidelines are shown according to the characteristics of each industry. Regarding ventilation, the specific rates or times are not indicated because of the various conditions of ventilation systems or windows.

Since pneumonia caused by SARS-CoV-2 was confirmed in December 2019, the mechanism of infection in an indoor environment has not been clarified. It is important to understand infection mechanism through various studies such as investigation of infection cluster spaces and experiments on the relationship between such environmental factors as temperature and humidity and the infectiousness of the virus. Based on these studies, further adequate strategy for the infection risk management in an indoor environment is required.

## Perspectives

In environmental stabilities of SARS-CoV-2, this virus can persist on the fomite surfaces, such as plastic or metal, between 3 and 7 days in an indoor environment such as a building. SARS-CoV-2 in aerosols is also stable for at least several hours if the virus is produced within small-particle aerosols. SARS-CoV-2 is mainly transmitted between humans through close contact and respiratory droplets, including fomite transmission. However, close-contact aerosol transmission through smaller aerosolized particles is likely to be combined with respiratory droplet and contact transmission in a confined, crowded, and poorly ventilated indoor environment, as some cluster cases have suggested. Therefore, adequate preventive measures to control indoor environmental quality are required. The risk of close-contact aerosol transmission can be reduced sufficiently by taking measures such as ensuring adequate ventilation in accordance with the number of individuals in a room and wearing masks to reduce the aerosols emitted into the indoor air. Filtration and UVGI systems have also been developed. However, indoor environmental quality control measures such as ventilation alone cannot prevent exposure from an infected person at a short distance or through environmental surfaces; thus, adequate preventive measures against respiratory droplet and contact transmission, such as disinfection of fomites, hand hygiene, uncrowded spacing, and the wearing of masks, must be taken at appropriate times and places.

In Japan, epidemiological links to confirmed COVID-19 cases showed that clusters of cases occurred in fitness gyms, a restaurant boat on a river, and a club with live music in February 2020. Based on these findings, the expert panel for COVID-19 focused on the “3 Cs,” namely, “Closed spaces with poor ventilation,” “Crowded spaces with many people,” and “Close contact.” In addition, the MHLW has recommended adequate ventilation in all closed spaces in accordance with the existing standard of the Law for Maintenance of Sanitation in Buildings. Although specific ventilation standards for the control of SARS-CoV-2 transmission cannot be derived from the existing scientific evidence, COVID-19 has caused serious global damage to public health, community, and the social economy. The United Nations Conference on Environment and Development adopted the Rio Declaration on Environment and Development in 1992 [111]. Principle 15 states: “In order to protect the environment, the precautionary approach shall be widely applied by states according to their capabilities. Where there are threats of serious or irreversible damage, lack of full scientific certainty shall not be used as a reason for postponing cost-effective measures to prevent environmental degradation.” This principle was also applied to the threats to human health in the public health domain [112–114]. Based on Principle 15, the implementation of practical precautionary measures that took into account technical feasibility, adequate control measures and strategies, and social, economic, and cultural conditions is essential to protect the public from SARS-CoV-2 transmission. However, the implementation of an approach based on the precautionary principle should start with a scientific evaluation, as complete as possible, and where possible, identifying at each stage the degree of scientific uncertainty [115]. Therefore, further research and evaluation are required to evaluate the effect and role of indoor environmental quality control, especially ventilation.

## Conclusions

In this paper, we summarize the effect and role of environmental factors in buildings, spatial dynamics, building operational factors, and a strategy to prevent SARS-CoV-2 transmission in a building environment. Most transmission has occurred in indoor environments in a closed space, especially inside a building. Although ventilation based on the existing regulation has been recommended by the MHLW as one of the initial political actions to prevent the spread of this novel infectious disease, the specific standard has not been recommended. To protect public health from SARS-CoV-2 transmission, further research on infection dynamics and the mode of infection in SARS-CoV-2 transmission in closed spaces, investigations on indoor environments in the infected cluster spaces, experiments on the influences of indoor environmental conditions on COVID-19 infection, and the effects of ventilation are urgently required. In addition, the development of control measures, guidelines, and strategies to maintain indoor environmental quality at an adequate level are required.

## Data Availability

Not applicable.
